# A Case of Undiagnosed Functional Gonadotroph Adenoma Leading to Ovarian Hyperstimulation Syndrome

**DOI:** 10.7759/cureus.26242

**Published:** 2022-06-23

**Authors:** Omkar Mayur, Ghada Elshimy, Rashika Bansal, Aasems Jacob, Rishi Raj

**Affiliations:** 1 Endocrinology, Diabetes, and Metabolism, Medical College of Georgia, Augusta University, Augusta, USA; 2 Endocrinology, Diabetes, and Metabolism, Cleveland Clinic, Cleveland, USA; 3 Hematology and Oncology, Pikeville Medical Center, Pikeville, USA; 4 Hematology and Oncology, Kentucky College of Osteopathic Medicine, University of Pikeville, Pikeville, USA; 5 Endocrinology, Diabetes, and Metabolism, Pikeville Medical Center, Pikeville, USA; 6 Endocrinology, Diabetes, and Metabolism, Kentucky College of Osteopathic Medicine, University of Pikeville, Pikeville, USA

**Keywords:** ovarian cyst, functional gonadotroph adenomas, ovarian hyperstimulation syndrome, follicle stimulating hormone, pituitary adenoma management

## Abstract

A functional gonadotroph adenoma is a very rare endocrinopathy, and only a few cases have been reported in the literature. We present a case of a woman in her early 50s with a past medical history of recurrent ovarian cysts who developed bilateral hemianopsia and was referred to the endocrinology clinic after a magnetic resonance imaging (MRI) identified a pituitary mass. Anterior pituitary hormone workup confirmed hypersecretion of follicle-stimulating hormone (FSH), which suggested ovarian hyperstimulation syndrome (OHSS) as the etiology of recurrent ovarian cysts. The patient underwent transsphenoidal resection of the pituitary tumor with improvement in visual symptoms. Our case illustrates that functional gonadotroph adenoma can be a potential cause of OHSS apart from the setting of assisted reproductive technology and hence warranting a meticulous endocrine evaluation to rule out this rare disease.

## Introduction

Functional gonadotroph adenomas (FGAs) are pituitary masses that secrete either follicle-stimulating hormone (FSH) or luteinizing hormone (LH) [1]. These are extremely rare endocrinopathies with only a few cases in the literature as the majority of immunohistochemically confirmed gonadotroph adenomas are nonfunctional [2]. While pituitary masses, in general, can cause symptoms of mass effect such as headaches and visual disturbances, FGAs can present with various ambiguous findings, such as menstrual disturbances, and gonadal stimulation, making them difficult to diagnose [2]. They are best managed with surgical excision, although options are available for medical management, they have no effect on tumor burden [2]. Ovarian hyperstimulation syndrome (OHSS) is most commonly an iatrogenic complication of assisted reproductive techniques. While its pathophysiology is largely unknown, the process is thought to be an amplified response to gonadotropin stimulation [3]. This article describes the case of an FSH-secreting pituitary adenoma presenting as OHSS in a premenopausal woman.

## Case presentation

A woman in her early 50s with a past medical history of recurrent ovarian cysts and hypothyroidism was referred to the endocrinology clinic for evaluation of a possible functioning pituitary macroadenoma found on an MRI of the brain. Six months prior to the presentation, the patient began experiencing bitemporal hemianopia. She also reported having right-sided frontotemporal headaches for the past several years but denied nipple discharge or trouble with conception and had a child. MRI brain done prior to presentation showed a 3.5 x 3.8 x 4.3 cm sellar and suprasellar mass consistent with a pituitary macroadenoma causing prechiasmatic optic nerve compression. Her medical history was significant for menorrhagia secondary to recurrent ovarian cysts for which she underwent total hysterectomy with bilateral oophorectomy 11 years prior to presentation. She also had hypothyroidism managed with a stable dose of levothyroxine 50 µg daily. On presentation to the endocrinology clinic, her vital signs were stable. Neurological examination confirmed bilateral temporal hemianopia, consistent with her history. 

**Figure 1:**
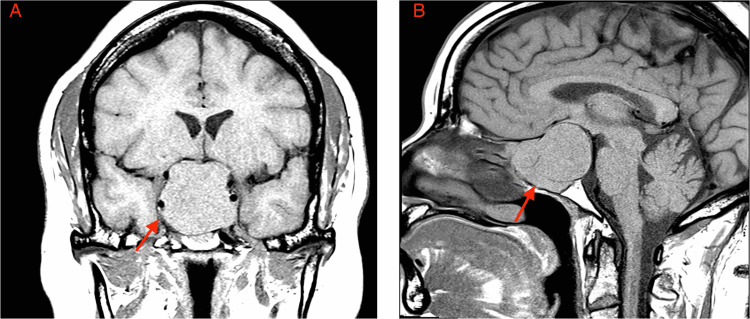
MRI of the brain MRI of the brain in the coronal section (A) and sagittal section (B) showed findings consistent with pituitary macroadenoma (red arrow) extending to the sphenoid sinuses and pterygoid recess, resulting in significant mass effect and atrophy of the right optic nerve.

**Figure 2:**
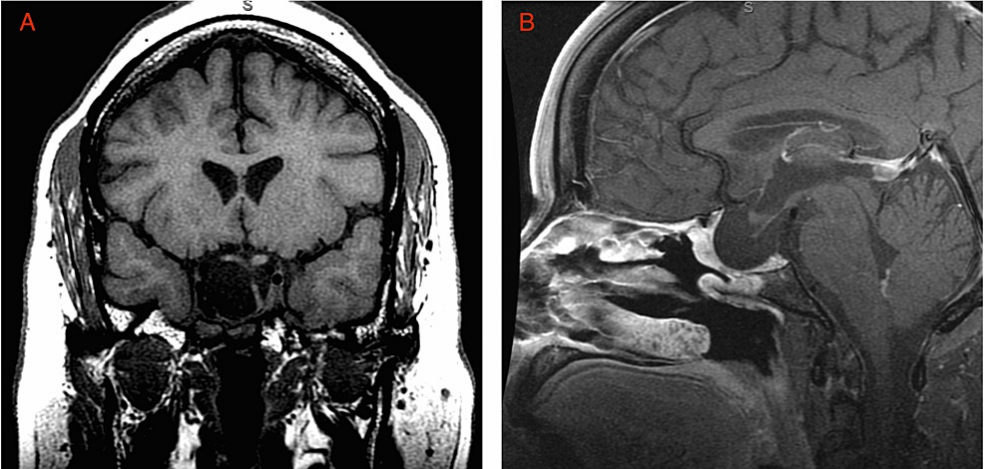
MRI of the brain MRI of the brain in the coronal section (A) and sagittal section (B) showed complete resection of the pituitary macroadenoma with postoperative changes.

Anterior pituitary hormone evaluation on presentation revealed elevated FSH and low LH (Table 1). The remaining labs were unremarkable. Based on the elevated FSH and history of recurrent ovarian cysts, it was determined that the mass identified on MRI was likely a true FSH secretory adenoma. Given the tumor size, mass effect, and lack of effective medical management, the patient was referred for surgery. A repeat MRI of the brain was obtained for surgical planning which confirmed the presence of a 4.1 x 3.6 x 3.9 cm mass extending into the sphenoid sinuses and pterygoid recess (Figures 1A, 1B). The patient underwent surgical debulking of the tumor by transsphenoidal resection two months following presentation, and histopathological examination confirmed FSH immunoreactive adenoma. Postoperatively, she was treated with hydrocortisone 30 mg daily, divided into a 20 mg and 10 mg dose, which was gradually tapered to discontinuation. A repeat pituitary panel following resection revealed a low FSH (2.55 mIU/mL) and LH level (0.75mIU/mL), indicating resolution of the FSH hypersecretion (Table 1). The patient had an uncomplicated hospital course and was discharged home three days after surgery. On follow-up two weeks after discharge, she reported a subjective improvement of 30% in her vision. At the three-month follow-up, she did not have further improvement in her visual symptoms and MRI confirmed gross total resection of the tumor with postsurgical changes (Figures 2A, 2B). Unfortunately, the patient was lost to follow-up afterward.

**Table 1:** Anterior pituitary hormone evaluation

Laboratory test	Results on presentation	Results postoperatively	Reference range
Thyroid-stimulating hormone (TSH)	2.877 mIU/mL	1.910 mIU/mL	0.6-4.7 mIU/mL
Free T4 (FT4)	0.81 ng/dL	1.10 ng/dL	0.58-1.76 ng/dL
Cortisol	8.91 µg/dL	-	5-15 µg/dL
Adrenocorticotropic hormone (ACTH)	25 pg/mL	-	7.2-63.3 pg/mL
Insulin-like growth factor (IGF-1)	60 ng/mL	54 ng/mL	54-258 ng/mL
Growth hormone (GH)	0.02 ng/mL	-	0.4-10 ng/mL
Prolactin	22.98 ng/mL	0.09 ng/mL	1.8-20.3 ng/mL
Luteinizing hormone (LH)	14.44 mIU/mL	0.75 mIU/mL	15.9-54.0 mIU/mL
Follicle-stimulating hormone (FSH)	186.83 mIU/mL	2.55 mIU/mL	23.0-116.3 mIU/mL

## Discussion

The incidence of pituitary adenomas is between 3.9 and 7.4 cases per 100,000 per year, and these tumors most commonly arise from gonadotroph cells, which account for 15%-40% [1,4]. These adenomas are generally nonfunctioning clinically but can cause headaches, visual disturbances, and hypopituitarism due to the mass effect [2]. In contrast, clinically functioning pituitary adenomas are extremely rare, accounting for less than 1% of all pituitary adenomas [1]. Functional gonadotroph adenomas (FGAs) can present in various ways depending on the patient, although many patients are asymptomatic [5]. Women often present with menstrual irregularities, and younger children may experience precocious puberty [2]. The excess secretion of FSH by a gonadotropin-secreting pituitary adenoma has also been associated with gonadal stimulation and OHSS in women of childbearing age [2]. 

OHSS itself is most often an iatrogenic complication of assisted reproductive techniques and results in enlarged ovaries with multiseptated cysts significantly larger than those found in polycystic ovary syndrome [2,3]. The pathogenesis is thought to be related to the ovarian response to FSH stimuli [3]. Conversely, patients who experience spontaneous OHSS should undergo further evaluation for an FGA [6].

In this case, the patient’s visual difficulties necessitated imaging, which identified the pituitary mass before any labs were obtained. However, in the absence of symptoms of mass effect, an elevated ratio of FSH to LH should raise suspicion for OHSS secondary to a pituitary adenoma, as FSH hypersecretion ultimately limits LH secretion via negative feedback [2]. In addition to elevated estradiol, most cases have a mild elevation of the prolactin level, likely due to pituitary stalk compression [2]. Our patient is unique as the diagnosis was likely missed in early reproductive age. She had the clinical characteristic including elevated FSH and low LH with a history of recurrent ovarian cysts further in addition to the adenoma seen on MRI. All of this indicates that the adenoma was not only functional but also symptomatic. It is also important to mention the difference between polycystic ovarian syndrome and OHSS as causes of ovarian cysts and we described the major clinic differences in Table 2 as it could sometimes lead to misdiagnosis of the patients [7,8].

**Table 2:** Comparison between PCOS and FSH-secreting adenoma with OHSS PCOS: polycystic ovarian syndrome, OHSS: ovarian hyperstimulation syndrome.

Investigations	PCOS	FSH-secreting adenoma with OHSS
Follicle-stimulating hormone (FSH)	Low	High
Luteinizing hormone (LH)	Elevated	Normal or low
Pelvic ultrasound	Mildly enlarged polycystic ovaries (cysts rarely >10 mm),	Grossly enlarged ovaries, with cysts measuring at least 15 mm)
Clinical hyperandrogenism	Usually, present	Unlikely

FGAs are generally treated with transsphenoidal resection of the tumor, which normalizes hormone levels, leads to regression of ovarian cysts, restores regular menses, and improves fertility [2]. For scenarios where surgical resection is difficult or contraindicated, medical therapy involves dopamine agonists, such as cabergoline [2]. These agents can reduce levels of FSH and estradiol and reduce the ovarian size but do not address the issue of tumor burden and mass effect [2]. Radiotherapy or radiosurgery may be also considered for residual tumors [1].

## Conclusions

Clinicians should have a high degree of suspicion for FSH-secreting pituitary adenoma while evaluating patients with multiple ovarian cysts who do not meet the criteria for PCOS especially if these patients are not undergoing any treatment with assisted reproductive technology. Early evaluation and diagnosis of the functional FSH-secreting pituitary adenoma could lead to the preservation of fertility and prevent patients from undergoing repeated surgeries for ovarian cyst removals and/or hysterectomy.

## References

[1] Wada-Hiraike O, Yamada S, Osuga Y (2022). An extremely rare case of pituitary functioning gonadotroph microadenoma accompanied by ovarian hyperstimulation syndrome in a woman of reproductive age. F S Rep.

[2] Patel S, Pacione D, Fischer I, Maloku E, Agrawal N (2019). Follicle-stimulating hormone-producing pituitary adenoma: a case report and review of the literature. AACE Clin Case Rep.

[3] Kumar P, Sait SF, Sharma A, Kumar M (2011). Ovarian hyperstimulation syndrome. J Hum Reprod Sci.

[4] Daly AF, Beckers A (2020). The epidemiology of pituitary adenomas. Endocrinol Metab Clin North Am.

[5] Garmes HM, Grassiotto OR, Fernandes YB, de Souza Queiroz L, Vassalo J, de Oliveira DM, Benetti-Pinto CL (2012). A pituitary adenoma secreting follicle-stimulating hormone with ovarian hyperstimulation: treatment using a gonadotropin-releasing hormone antagonist. Fertil Steril.

[6] Castelo-Branco C, del Pino M, Valladares E (2009). Ovarian hyperstimulation, hyperprolactinaemia and LH gonadotroph adenoma. Reprod Biomed Online.

[7] De Croos M, Vender J, Elshimy G, Stachura M (2021). A follicular stimulating hormone secreting adenoma. J Endocr Soc.

[8] Kawaguchi T, Ogawa Y, Ito K, Watanabe M, Tominaga T (2013). Follicle-stimulating hormone-secreting pituitary adenoma manifesting as recurrent ovarian cysts in a young woman: latent risk of unidentified ovarian hyperstimulation: a case report. BMC Res Notes.

